# From plant surface to plant metabolism: the uncertain fate of foliar-applied nutrients

**DOI:** 10.3389/fpls.2013.00289

**Published:** 2013-07-31

**Authors:** Victoria Fernández, Patrick H. Brown

**Affiliations:** ^1^Forest Genetics and Ecophysiology Research Group, School of Forest Engineering, Technical University of MadridMadrid, Spain; ^2^Department of Plant Sciences, University of California at Davis, DavisCA, USA

**Keywords:** apoplast, cuticle, foliar fertilizers, foliar uptake, leaf, foliar sprays, nutrient mobility, plant surfaces

## Abstract

The application of agrochemical sprays to the aerial parts of crop plants is an important agricultural practice world-wide. While variable effectiveness is often seen in response to foliar treatments, there is abundant evidence showing the beneficial effect of foliar fertilizers in terms of improving the metabolism, quality, and yields of crops. This mini-review is focused on the major bottlenecks associated with the uptake and translocation of foliar-applied nutrient solutions. A better understanding of the complex scenario surrounding the ultimate delivery of foliar-applied nutrients to sink cells and organs is essential for improving the effectiveness and performance of foliar fertilizers.

## FOLIAR NUTRIENT UPTAKE AND PLANT RESPONSE TO THE TREATMENTS: A COMPLEX SCENARIO

Foliar fertilization is an important tool for the sustainable and productive management of crops, and is of significant commercial importance world-wide. The rationale for the use of foliar fertilizers include: (1) when soil conditions limit availability of soil applied nutrients; (2) in conditions when high loss rates of soil applied nutrients may occur; (3) when the stage of plant growth, the internal plant demand and the environmental conditions interact to limit delivery of nutrients to critical plant organs. In each of these conditions, the decision to apply foliar fertilizers is determined by the magnitude of the financial risk associated with the failure to correct a deficiency of a nutrient and the perceived likelihood of the efficacy of the foliar fertilization. Our current understanding of the factors that influence the ultimate efficacy of foliar nutrient applications is, however, incomplete (Kannan, 2010; Noack et al., 2010; Fernández et al., 2013).

Many factors influence the performance of foliar nutrient sprays, but for simplicity they may be grouped under physicochemical properties of the formulation, the environment under which sprays are applied or the characteristics of the plant to which the spray is applied. Physico-chemical properties of the spray formulation such as molecular size, solubility, or electric charge, pH, surface tension, retention, spreading, or point of deliquescence of the formulations all play a major role in determining the efficacy of uptake of nutrient solutions by the foliage (Fernández and Eichert, 2009; Fernández et al., 2013). Environmental factors affect the uptake and translocation of foliar nutrient sprays by influencing both plant response and the properties of the formulation and include relative humidity, temperature, and light (Fernández and Eichert, 2009; Fernández et al., 2013). Finally, efficacy is influenced by the physiological status of the plant and species characteristics including leaf shape, leaf chemistry, and physical attributes including cuticle composition, surface wax architecture, the presence of leaf hairs, leaf surface architecture, phenological stage, the mobility of the nutrient within the plant, or the presence of abiotic stresses. All of these factors interact to alter the absorption and translocation of foliar nutrient sprays and ultimately the plant response (Fernández et al., 2013). A brief account of the major plant physico-chemical, anatomical, and physiological barriers influencing the rate of uptake and translocation of foliar-applied nutrients is provided in the following paragraphs.

## CROSSING THE PLANT SURFACE

The permeability of plant surfaces to nutrients dissolved in water has been studied for more than a century in parallel with studies of plant surface composition, structure, and function (Fernández and Eichert, 2009). Scientific progress during the last decades has provided a better, though incomplete, understanding of the processes affecting plant responses to foliar nutrient applications and has led to an increased use of this fertilization strategy in agriculture (Fernández et al., 2013).

The epidermal cells of most aerial plant surfaces (e.g., of fruits, leaves, flowers, or stems) are covered with an extra-cellular layer, the cuticle, which is the interface between the plant organs and the surrounding environment. The cuticle protects plant organs against multiple biotic and abiotic stress factors, and is crucial for minimizing water loss (Kerstiens, 1996). This generally lipid-rich protecting layer is chiefly made of a biopolymer matrix of cutin and/or cutan, with waxes deposited on to and intruded into it, in addition to variable amounts of polysaccharides and phenolics (Domínguez et al., 2011). The chemical composition and structure of the cuticle of most plant species and organs remains unclear (Khayet and Fernández, 2012), and has been found to vary in response to environmental and physiological conditions during growth and development (Kosma et al., 2009; Domínguez et al., 2011), therefore limiting the applicability of individual studies to other species or conditions.

In addition to cuticle production, the epidermis of plants contains specialized cells including trichomes or stomata that may influence foliar nutrient uptake. A major degree of plant surface heterogeneity and different nano- and/or micro-scale levels of epidermal cell, epicuticular wax, and cuticular sculpturing have been observed by scanning electron microscopy (SEM; Koch and Barthlott, 2009). Some plant materials have been found to have a high surface roughness, which may lead to an increased hydrophobicity (Koch and Barthlott, 2009; Konrad et al., 2012).

The commercial significance of foliar sprays of plant protection products, herbicides, fertilizers, or plant growth regulators for agricultural production, has resulted in many cuticular permeability trials over the past 60 years (Riederer and Friedmann, 2006; Fernández and Eichert, 2009; Schreiber and Schönherr, 2009). Such studies enabled the development of the “dissolution–diffusion model” for the cuticular penetration of apolar, lipophilic compounds (Riederer and Friedmann, 2006). In contrast, the mechanisms of penetration of hydrophilic, polar solutes through the cuticle are currently not fully understood (Fernández and Eichert, 2009).

For at least some plant species, there is now clear evidence for the stomatal uptake of water and solutes in the absence of an external pressure or surface-active ingredients (Eichert et al., 1998, 2008; Burkhardt et al., 2012). The overall contribution of stomata to the foliar uptake process, however, remains unclear, but can be highly significant (Eichert et al., 2008), and may vary according to factors such as plant species and variety, leaf phenological stage, and stomatal functionality and density, or due to the prevailing environmental conditions during plant growth and development. Solutes penetrating stomata have been suggested to follow a diffusion pathway along the pore walls, which appears to be less size selective than in the cuticle (Eichert et al., 2008). The role of epidermal structures such as trichomes or lenticels in the absorption of surface-applied nutrient solutions is currently unclear (Fernández et al., 2013).

**FIGURE 1:**
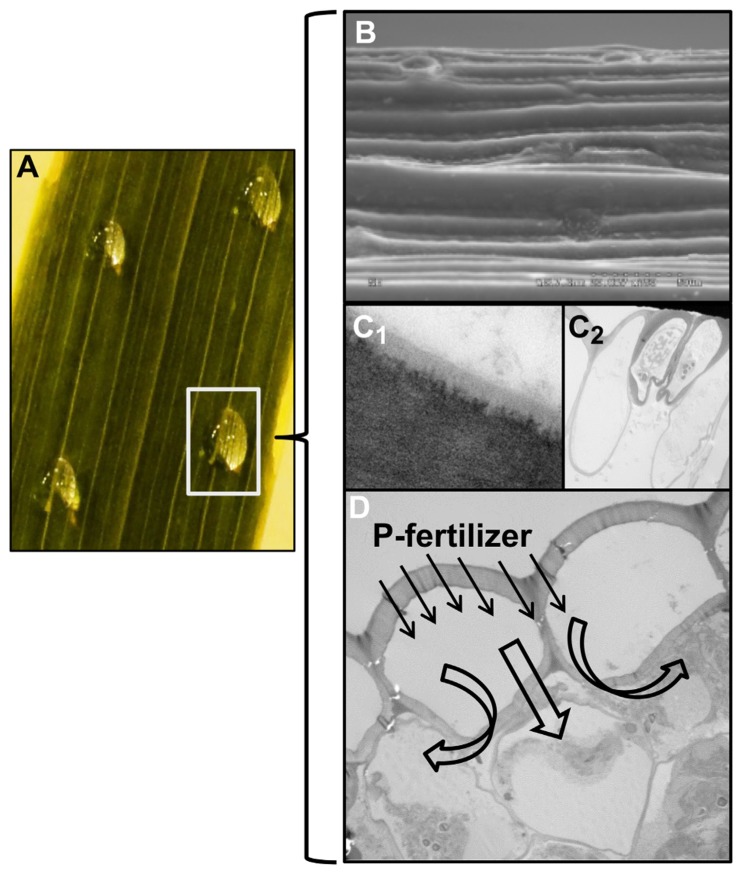
**Major factors affecting the absorption and mobility in plant tissues of nutrient solutions applied on to plant surfaces.** An example is provided with images corresponding to the application of P fertilizer drops on to the abaxial surface of a wheat leaf **(A)**. The interactions between fertilizer solution drops and the wheat leaf surface (**B**; SEM micrograph) will influence foliar P uptake via the cuticle **(C_1_**) or stomatal pores **(C_2_**). Phosphorus may be subsequently transported in the apoplast, taken up by epidermal cells and translocated to other plant parts **(D)**. Figures **(C,D)** are transmission electron micrographs of the abaxial surface of a wheat leaf.

Recently, Khayet and Fernández (2012) highlighted the relevance of considering the combined effects of the physical structure, polarity, and hydrophobicity of plant surface constituents and surface deposited liquids (e.g., solutes and solvents of agrochemical treatments) or materials (e.g., foliar-applied agrochemicals or aerosols), when analyzing plant surface interactions. Such phenomena are key since they are a preliminary step in the absorption of foliar fertilizers (**Figure 1A**). When applying drops of a 2% phosphorus (P)-containing solution on to the abaxial surface of a wheat leaf, the liquid will interact with the leaf surface, giving rise to a specific contact angle and work-of-adhesion (Fernández et al., 2011). The droplet–leaf interaction will depend on the physico-chemical characteristics of the spray solution (e.g., surface tension, polarity, or hydrophobicity) and of the plant surface (i.e., the combined effects of roughness and chemical composition; **Figure 1B**). The higher the contact area between the fertilizer drops and the plant surface, the greater will be the chance for uptake to occur via the cuticle (**Figure 1C_1_**) or stomatal pores (**Figure 1C_2_**). The rate of foliar spray retention or repulsion will depend on the interactions between the fertilizer drops and plant surfaces, and the same fertilizer formulation may perform differently when applied on to different plant species, varieties, or organs. For example, Picchioni et al. (1995) measured the rate of retention of foliar-applied boric acid (1 g L^-^^1^ plus 0.05% Triton X-100) and observed that apple leaves retained four times the volume on an area basis than sweet cherry leaves, and about twice the volume of fertilizer retained by prune and pear leaves.

While the mechanisms of absorption of water and solutes through the cuticle and stomata are not fully characterized, the affinity (solubility) of polysaccharides for aqueous solutions is higher than that of waxes and cuticle matrix bio-polymers (Khayet and Fernández, 2012). Most active ingredients are based on salts that will ionize when dissolved in water. While many metal chelates are negatively charged and a few compounds like urea or boric acid may be neutral (Picchioni et al., 1995), most nutrient salts will have positive charges and may potentially bind to the existing free carboxyl and hydroxyl groups present in the cuticle, which will act as an ion exchange membrane. This phenomenon may be even stronger when considering the diffusion of nutrients across the cell walls, which is a pre-requisite for the translocation of foliar-applied mineral elements from the point of application to different plant parts (**Figure 1D**).

## LOST IN SPACE: MOBILITY OF FOLIAR-APPLIED NUTRIENTS IN THE APOPLAST

The apoplast is defined as the area within the plant tissues which is beyond the cell plasma membrane, and includes the cell wall, middle lamella, xylem, and gas and water filled intercellular spaces (Sattelmacher, 2001). Hence, while the internal border of the leaf apoplast is the cell plasmalemma, its outer limit is the cuticle which lays over the epidermal cell wall. The interaction of foliar-applied nutrients with the apoplast has not been examined, however, given the role of the apoplast in ion balance (Grignon and Sentenac, 1991) and element detoxification (Sattelmacher, 2001), the transmission of signals leading to stomatal responses (Fujita et al., 2013), and regulation of transient pH fluctuations (Geilfus and Mühling, 2011), it is reasonable to expect that characteristics of the apoplast will influence the fate of foliar-applied nutrients.

The leaf apoplast plays a role in ion exchange and as a diffusion barrier, and may accumulate cations and repel anions as reported in several investigations (Speer and Kaiser, 1991; Sattelmacher, 2001; White and Broadley, 2011). Estimates of the volume of leaf water in the apoplast vary from 10 to 35% of total leaf water (Speer and Kaiser, 1991; Wardlaw, 2005). Due to the predominant functional groups of the polysaccharides forming the primary cell wall and middle lamella (Khayet and Fernández, 2012), a higher degree of polar and hydrogen-bonding interactions with water and solutes can be expected in the apoplastic space as compared to the cuticle. Ions present in the apoplast may also induce ionic shifts and changes in polysaccharide material properties (Lee et al., 2012).

While there is little information on the direct fate of foliar-applied nutrients in the leaf apoplast, restrictions to the mobility of elements supplied as cations such as zinc (Zn), iron (Fe), or calcium (Ca) can be expected due to the abundance of negative charges in the apoplastic space, which may limit their translocation to other plant compartments and/or organs.

Fernández et al. (2005) suggested that foliar application of non-charged or electron-charged Fe chelates may penetrate the leaf and to be translocated in the apoplast more readily than positively charged or ionic Fe-containing substances. Results demonstrate, however, that there is limited mobility of all Fe compounds even within the treated leaf (e.g., Fernández et al., 2005, 2008), and that this may vary with the combination of multiple factors such as plant species, organ ontogeny, or mineral element compound.

Recent evidence suggests that interactions of nutrients with the apoplast can play a critical role in plant development processes (De Freitas et al., 2012). Ca deficiency is a common problem affecting horticultural commodities and the application of Ca sprays is a widespread practice, which may induce variable plant responses (Fernández et al., 2013). De Freitas et al. (2012) clarified the mechanisms of blossom end rot development in tomato fruit, and observed that high levels of pectin methylesterase production increased the level of free carboxylic acids in cell walls, which will bind Ca and make it unavailable for other metabolic processes.

In summary, while very little is known about the movement and interaction of foliar-applied nutrients in and through the apoplastic space, factors within the apoplast including cell wall charge, pore size, pH, ionic strength, chemical form in which the nutrients are supplied and water fluxes in the apoplast have a clear potential to alter element mobility and the subsequent translocation to different plant parts. Improving our understanding of the underlying phenomena associated with the apoplastic movement of foliar-applied nutrients will be essential to the optimization of foliar fertilizer strategies.

## THE UNCERTAIN PATHWAY FROM SYMPLAST OF SINK CELLS AND ORGANS

The efficacy of foliar nutrient sprays may additionally depend on their ability to be transported to other plant organs including fruits, grains, young leaves, or flower, which may vary in relation to different plant species and varieties, plant phenology, sprayed organ ontogeny, or environmental conditions during treatment, and therefore cannot be generalized (Fernández et al., 2013). In relation to their phloem mobility, essential nutrients have been classified as highly mobile (N, P, K, Mg, S, Cl, Ni), intermediate or conditionally mobile (Fe, Zn, Cu, B, Mo), and rather immobile (Ca, Mn; Marschner, 2012). Hence, foliar sprays of elements with a higher mobility are theoretically more likely to induce systemic responses in plants in contrast to the local effect of the immobile nutrients.

This implies that foliar fertilizer effectiveness may sometimes be interpreted in terms of their benefit to local or whole plant processes and in relation with nutrient mobility, which among other factors, may be affected by plant species and varieties, or organ ontogeny. For instance, in many species most Zn, Mn, Ca, and Fe sprays are local in their effect with only very limited transport out of the sprayed leaf tissues (Zhang and Brown, 1999; Fernández et al., 2013). Nevertheless, such treatments may still have a significant local benefit, and even a relatively small transport out of treated leaves may have a short-term but critical advantage to the plant.

Recent evidence suggests that the remobilization of foliar Zn (both soil-derived and foliar-applied) is influenced by plant species and genotypes, phenological stage, application method, or the prevailing environmental conditions (Kutman et al., 2010; Impa et al., 2013). Whether foliar-applied or soil-derived nutrients behave differently is currently unknown, similarly there is no clear consensus that the form of the applied foliar nutrient influences the metabolic efficacy of the absorbed nutrient.

**FIGURE 2:**
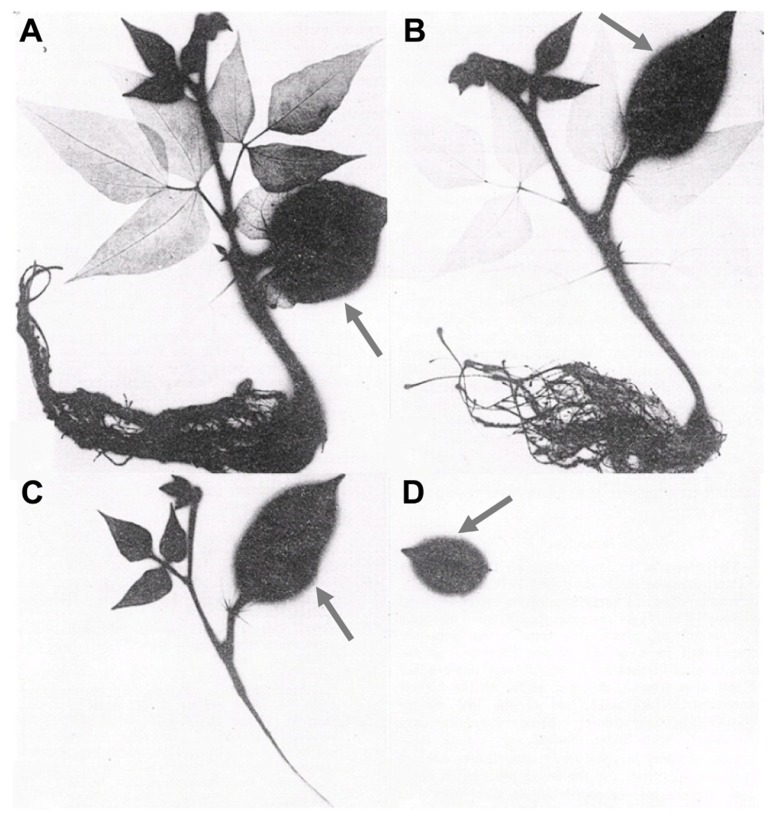
**Translocation of foliar-applied ^**32**^P when supplied to leaves of different maturity stages of bean seedlings (Koontz and Biddulph, 1957)**. Radiolabeled P was applied to the indicated leaf (arrow) by immersion of mature leaves **(A,B)**, younger leaves **(C)**, and immature leaves **(D)**. The distribution of labeled P was visualized 24 h after foliar application.

While many aspects related to the mobility of foliar-applied nutrients remain unclear (Fernández et al., 2013), leaf development is clearly an important factor that will influence nutrient export from and import into leaves and other organs (Turgeon, 2006). As leaves develop they transition from sink organs that are entirely dependent upon imported assimilate to source organs that export nutrients to other organs in the plant. Immature leaves are physiologically incapable of exporting nutrients until they have matured, while old leaves are incapable of importing nutrients once they have reached maturity. This phenomenon is illustrated in **Figure 2**, in which bean leaves of sequentially younger age were treated with radioactive P (^32^P). The resultant translocation of ^32^P is represented by the dark areas in the autoradiograph imaged 24 h after ^32^P application (Koontz and Biddulph, 1957).The application of ^32^P to mature leaves (**Figures 2A,B**) resulted in rapid transport of the ^32^P-containing products to young developing leaves and roots with no transport into mature leaves. With ^32^P application to successively younger leaflets (**Figure 2C**), transport out of the treated leaf was reduced and restricted to the nearest sink tissue (apical shoot meristems) with no ^32^P transported to the root. Application of ^32^P to immature leaves (**Figure 2D**) resulted in 100% retention of ^32^P in the treated leaf. While the timing with which leaves transition from sink to source varies between species and environments, the effect of this transition on the ability of leaves to export foliar-applied nutrients is a general principle that should be considered when designing and interpreting foliar fertilizer applications.

## CONCLUDING REMARKS

The complexity of factors that govern the efficacy of foliar nutrient spray can be illustrated by considering the performance of mineral elements and compounds known to have different leaf absorption rates and mobility within plant tissues and organs (Fernández et al., 2013). Foliar-applied urea solutions are highly permeable and the resultant N metabolites are easily transported from mature leaves to sink organs (Bondada et al., 2001; Stiegler et al., 2011). Boron compounds in contrast can be absorbed by leaves at rates almost equivalent to urea, but have limited mobility within many plant species making their efficacy strictly local (Brown and Shelp, 1997; Brown et al., 2002; Will et al., 2011). In species with high phloem B mobility, foliar B application results in rapid absorption and rapid movement of B to sink tissues (Brown and Shelp, 1997). Finally, both the foliar uptake and the translocation of Zn are highly limited in pistachio and walnut and hence the benefit of Zn sprays is entirely localized to the organs that directly received the application (Zhang and Brown, 1999). In wheat and likely in other small grain species, significant Zn mobility can occur but is strongly dependent on factors such a plant nutritional status, species and variety, or plant phenological state (Wu et al., 2010; Erenoglu et al., 2011; Hegelund et al., 2012; Kutman et al., 2012). These examples illustrate the diversity of plant response to foliar fertilizers. Improving the efficacy and utility of foliar fertilizers will require a sound understanding of the physical, chemical, biological, and environmental principles that govern the absorption, translocation, and utilization of foliar-applied nutrients by plants.

## Conflict of Interest Statement

The authors declare that the research was conducted in the absence of any commercial or financial relationships that could be construed as a potential conflict of interest.
